# A Treatise on the Practice of Medicine for the Use of Students and Practitioners of Medicine

**Published:** 1887-06

**Authors:** 


A TREATISE ON THE PRACTICE OF MEDICINE FOR THE USE OF STUDENTS AND PRACTITIONERS OF MEDICINE. By Roberts Bartholow, M. A., M. D., LL. D. New York: D. Appleton & Co. 1886.

Dr. Bartholow's book has gone through six editions in six years. This furnishes incontrovertible evidence that it is popular with a very large class of readers. As it has been before the profession so long, any extended criticism of it is superfluous. The present edition has had a number of new subjects added to it.

To the young practitioner, Dr. Bartholow's work is very apt to prove disappointing; he will find under the treatment of each disease a number of sovereign remedies, the author being somewhat of a "therapeutical optimist," but, alas, in actual practice these remedies will not "work." The author's wide-spread reputation and his wonderfully clear method of expression will always secure for his book a large patronage. H. P. C.

